# Hepatocellular carcinoma with extension to the diaphragm, falciform ligament, rectus abdominis and paraumbilical vein

**DOI:** 10.2349/biij.4.4.e37

**Published:** 2008-10-01

**Authors:** R Kaur, BJJ Abdullah, V Rajasingam

**Affiliations:** 1 Department of Biomedical Imaging, University of Malaya Medical Centre, Kuala Lumpur, Malaysia; 2 Department of Surgery, University of Malaya Medical Centre, Kuala Lumpur, Malaysia

**Keywords:** Hepatocellular carcinoma, metastases, computed tomography

## Abstract

Hepatocellular carcinoma is the most common primary tumour of the liver. The most common extrahepatic metastatic sites are the lung, lymph nodes, bones and adrenal glands. All forms of HCC demonstrate a tendency for vascular invasion, producing extensive intrahepatic metastases and, occasionally, portal vein or inferior vena cava extension with spread into the right atrium in extreme cases. Tumour spread of abdominal diseases via hepatic ligaments has also been previously reported. We report a rare case of hepatocellular carcinoma with extension into the falciform ligament, overlying rectus sheath and adjacent diaphragm with concomitant infiltration into the recanalised paraumbilical vein.

## CASE REPORT

A 73 year-old male presented to our centre with a painful swelling in the epigastric region which progressively increased in size over 1 month, alongside loss of appetite and fever with chills. He was an alcoholic, diabetic and also suffered from ischaemic heart disease. There was no history of hepatitis or prior blood transfusion. Physical examination revealed a firm, well-defined erythematous but smooth-surfaced epigastric mass measuring 8×7 cm. Spider naevi was noted on the legs and jaundice in the left sclera (right eye was blind).

Pertinent laboratory values were as follows: white blood cell count 10.1×10^9^ /L (normal 4-11); haemoglobin 103.0 g/L (normal 130.0-180.0); haematocrit 0.34 (normal 0.4-0.5); total protein 54 g/L (normal 64-82); albumin 24 g/L (normal 35-50); total bilirubin 8 μmol/L (normal 3-17); ALT 77 IU/L (normal 30-65); AST 83 IU/L (normal 15-37); α-fetoprotein 9 IU/L; Hepatitis B and C profile was negative and coagulation profile was normal.

**Figure 1:**
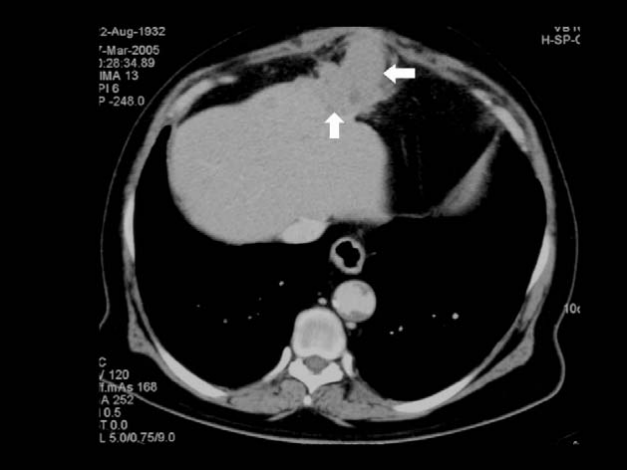
Scan performed in the late arterial phase demonstrating HCC (arrows) arising from liver with surrounding inflammatory changes, obliteration of the fat plane between the tumour mass and rectus sheath with extension into the overlying anterior abdominal wall.

**Figure 2:**
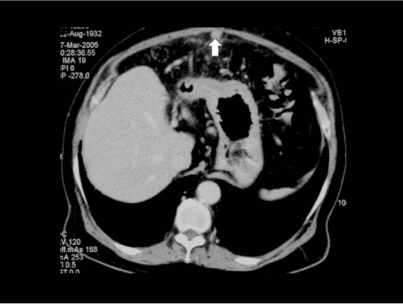
Scan performed in the late arterial phase demonstrating tumour infiltration into the recanalised paraumbilical vein (arrow).

An ultrasound examination revealed an ill-defined hypoechoic mass measuring 5.7×7.4 cm in the left lobe of the liver. The biliary ducts, gallbladder, pancreas and spleen were normal. Vascular channels ie. inferior vena cava, portal and hepatic veins were also patent. A provisional diagnosis of liver tumour was made. Subsequently a CT scan revealed an exophytic mass arising from an atrophic left lobe of the liver with surrounding inflammatory changes and extension into the overlying anterior abdominal wall (Figure 1) with maximum enhancement during the delayed arterial phase (scan delay 35 s). The fat plane between the tumour mass and the rectus sheath was obliterated and the tumour extended superiorly from the level of the right ventricle to the fundus of the stomach inferiorly. It measured 7.2×8.0×5.1 cm. The tumour was also noted to extend into the recanalised paraumbilical vein (Figure 2). Liver was nodular in outline and the left lobe was atrophic in keeping with cirrhosis. Spleen and portal vein was normal. There were no peritoneal seedlings, lymphadenopathy, lung nodules or bony lesions to suggest metastases.

Liver biopsy revealed a well-differentiated hepatocellular carcinoma. Patient underwent a wedge resection of segment 2. Intraoperative findings revealed an 8 cm nodular exophytic tumour arising from segment 2 of the liver with extension to the anterior abdominal wall and undersurface of the left hemidiafragm. The surface of the liver was nodular in appearance. Patient also underwent a cholecystectomy and excision of the infiltrated left diaphragm.At histopathology the specimen showed a tumour composed of nodules of malignant cells in sheets with moderate vascularity. The cells appeared hepatoid and displayed large irregular hyperchromatic vesicular nuclei and prominent nucleoli. Fibrous septa separated the tumour nodules and there was a thin capsule around the tumour. The adjacent hepatic tissue showed portal tract infiltration by chronic inflammatory cells. Special stains showed that immunohistochemistry for HBV was negative. The diaphragmatic specimen consisting of skeletal fibres showed nodules of malignant cells similar to that described in the liver specimen. There was no gallbladder invasion.

## DISCUSSION>

The most important risk factor for the development of hepatocellular carcinoma (HCC) appears to be chronic hepatitis B or C viral infection [1]. Liver cirrhosis has also been identified in over 70% of HCC patients in the Western countries and has been identified as a predisposing factor.

Many different treatment options are available depending on the staging of this aggressive tumour [2]. The commonest extrahepatic metastatic sites are the lung, lymph nodes, bones and adrenal glands [2,3]. Less common metastatic sites include the brain, bladder, gastrointestinal tract, diaphragm, seminal vesicles and pancreas [2]. All forms of HCC demonstrate a tendency for vascular invasion, producing extensive intrahepatic metastases and, occasionally, portal vein or inferior vena cava extension with spread into the right atrium in extreme cases [4]. Although these vessels were intact in our patient, vascular invasion was demonstrated by the presence of tumour within the recanalised paraumbilical vein.

Kim et al. and Mori et al. [5,6] have previously reported cases of HCC extending into rectus abdominis via ligamentum teres. Meyers et al. [7,8] initially established the concept of tumour spread of abdominal diseases via hepatic ligaments.

Radiologically, disease processes, such as fluid collections within and obliterating the perihepatic-ligamentous fat or focal collections near the ligamentous attachments suggests ligamentous spread. Concommitantly there may be continous spread, also suggesting ligamentum continuity on CT scans or sonograms [6]. Ligamentous spread of the tumour was clearly demonstrated by the presence of sorrounding inflammatory changes, extension into the overlying anterior abdominal wall and obliteration of the fat plane between the tumour mass and rectus sheath. Unfortunately these findings were not well appreciated on ultrasound examination.

We therefore postulate the extension into the anterior abdominal wall may have occurred by direct extension of the exophytic tumour component into the paraumbilical vein within the round ligament and rectus via the falciform ligament. In addition, this patient had no history of having undergone a liver biopsy prior to discovery of the tumour mass, excluding tumour seeding along a previous biopsy tract.

Although the HPE specimen analysis did not reveal evidence of fibrosis or cirrhosis of the liver parenchyma adjacent to the tumour, a thin capsule was found around the tumour and the nodular surface of the liver was confirmed macroscopically during surgery.

A curative surgical approach with wedge resection of segment 2 of the liver was performed as the tumour was found to be confined to segment 2 with no evidence of metastases. The patient was subsequently followed up clinically at 3-monthly intervals with 6-monthly follow-up CT scans. A year after the operation, the patient developed tumour recurrence at the operative scar site on the anterior abdominal wall. He underwent a single cycle of radiotheraphy and subsequently chemotheraphy; however the tumour failed to respond favourably. The tumour demonstrated a slow growth pattern and currently measures approximately 3×4 cm. The patient is currently being managed conservatively and is otherwise well.
